# Supplementary material for: Geographic patterns of distribution and ecological niche of the snake-necked turtle genus Hydromedusa
Source: PeerJ. 2024 Mar 26;12:e16712. doi: 10.7717/peerj.16712 (PMC10979749; doi:10.7717/peerj.16712)
Supplement: Table S2 [file peerj-12-16712-s002.docx]

Table S2: Range of climatic variables to *Hydromedusa* species.

| **Species** | **Climatic variable** | **BIO** | **Range** |
| --- | --- | --- | --- |
| *H. tectifera* | Temperature Seasonality | 4 | 146–6678 |
| *H. tectifera* | Precipitation of Warmest Quarter | 18 | 3–35700 |
| *H. tectifera* | Mean Diurnal Range | 2 | 4–176 |
| *H. maximiliani* | Temperature Seasonality | 4 | 137–7083 |
| *H. maximiliani* | Isothermality | 3 | 0.8–8.72 |
